# Chitinase Chit62J4 Essential for Chitin Processing by Human Microbiome Bacterium *Clostridium paraputrificum* J4

**DOI:** 10.3390/molecules26195978

**Published:** 2021-10-02

**Authors:** Jan Dohnálek, Jarmila Dušková, Galina Tishchenko, Petr Kolenko, Tereza Skálová, Petr Novák, Karla Fejfarová, Jiří Šimůnek

**Affiliations:** 1 Laboratory of Structure and Function of Biomolecules, Institute of Biotechnology of the Czech Academy of Sciences, v. v. i., Biocev, Průmyslová 595, 252 50 Vestec, Czech Republic; duskovaj@ibt.cas.cz (J.D.); petr.kolenko@ibt.cas.cz (P.K.); tereza.skalova@ibt.cas.cz (T.S.); karlafej@gmail.com (K.F.); 2 Department of Structural Analysis of Biomacromolecules, Institute of Macromolecular Chemistry of the Czech Academy of Sciences, v. v. i., Heyrovsky Sq. 2, 162 06 Prague, Czech Republic; gtiscenko@gmail.com; 3 Laboratory of Structural Biology and Cell Signaling, Institute of Microbiology of the Czech Academy of Sciences, v. v. i., Biocev, Průmyslová 595, 252 50 Vestec, Czech Republic; pnovak@biomed.cas.cz; 4 Laboratory of Anaerobic Microbiology, Institute of Animal Physiology and Genetics of the Czech Academy of Sciences, v. v. i., Vídeňská 1083, 142 00 Prague, Czech Republic; juraseksimunek@seznam.cz

**Keywords:** human commensal bacterium, chitinase, exochitinase, glycosyl hydrolase family 18, adaptation to the environment

## Abstract

Commensal bacterium *Clostridium paraputrificum* J4 produces several extracellular chitinolytic enzymes including a 62 kDa chitinase Chit62J4 active toward 4-nitrophenyl *N*,*N*′-diacetyl-β-d-chitobioside (pNGG). We characterized the crude enzyme from bacterial culture fluid, recombinant enzyme rChit62J4, and its catalytic domain rChit62J4cat. This major chitinase, securing nutrition of the bacterium in the human intestinal tract when supplied with chitin, has a pH optimum of 5.5 and processes pNGG with *K*_m_ = 0.24 mM and *k*_cat_ = 30.0 s^−1^. Sequence comparison of the amino acid sequence of Chit62J4, determined during bacterial genome sequencing, characterizes the enzyme as a family 18 glycosyl hydrolase with a four-domain structure. The catalytic domain has the typical TIM barrel structure and the accessory domains—2x Fn3/Big3 and a carbohydrate binding module—that likely supports enzyme activity on chitin fibers. The catalytic domain is highly homologous to a single-domain chitinase of *Bacillus cereus* NCTU2. However, the catalytic profiles significantly differ between the two enzymes despite almost identical catalytic sites. The shift of pI and pH optimum of the commensal enzyme toward acidic values compared to the soil bacterium is the likely environmental adaptation that provides *C. paraputrificum* J4 a competitive advantage over other commensal bacteria.

## 1. Introduction

Bacteria in the large intestine are one of the most diverse microbial communities in nature [1]. These microbial communities rely on digestible proteins, fats, and poly- and oligosaccharides as an energy source as well as on non-digestible polysaccharides reaching the colon. The ability to utilize non-digestible polysaccharides plays an important role in maintaining the equilibrium of individual bacterial species in the mammalian microbiome. This has an impact through microbial ecology on human health [2]. Most non-digestible polysaccharides originate from plants and other natural foods, but a significant proportion come from insects or artificial chitin-based or chitin-derived nutrition, among other sources [3]. The occurrence of commensal bacterial species capable of chitin degradation illustrates the adaptation of the human microbiome to the environment and nutrition development or variation [4]. Convergence of enzymatic functions of carbohydrate-active genes under environmental pressure in human gut microbes was first illustrated in glycoside hydrolases and glycosyltransferases, with horizontal gene transfer and parallel gene loss being the expected primary mechanisms [5].

Gram-positive bacteria with low GC content form the most abundant bacterial group of the intestinal microbiome [1]. The distribution of individual microbiome species along the human colon is not uniform but is influenced by several factors. According to [6], the change of pH along the human gut may be responsible for a major drift within the human fecal bacterial community. In particular, the growth of a major group of Gram-negative bacteria is inhibited at mildly acidic pH, thus creating space for a lower pH-tolerant microorganism. Clostridial clusters of bacteria are significantly more abundant at pH 5.5 compared to pH 6.5. However, when considering chitin digestion, colonization of insoluble polysaccharide fibers in the intestine may have a more complex pattern with respect to pH and its local variation, although detailed knowledge in this area is missing. However, a bacterium able to tolerate lower pH, typical for the proximal parts of the human colon, while digesting chitin or its derivatives, would have an advantage in competition with other intestinal microflora under specific conditions.

Particular adaptations of human gut microbial enzymes under highly competitive microbiome conditions at the level of enzyme structure and function have been given little attention. However, one example is a study of α-glucosidase from *Ruminococcus obeum*, capable of producing glucose from both isomaltose and maltose [7].

Chitinases (E.C. 3.2.1.14) are enzymes cleaving the β-1,4 bond of the natural polysaccharide chitin, composed of *N*-acetyl-d-glucosamine units (GlcNAc)n. Bacterial chitinases have been studied and utilized for biotechnological purposes [8,9]. Chitinases belong to glycosyl hydrolase (GH) classes, GH18, 19, 23 and 48 [10,11]. The GH18 family contains archaeal, bacterial, eukaryotic, and viral chitinases, family 19 only bacterial and eukaryotic ones, and family 48 mainly eukaryotic ones. Chitinases of the GH18 family perform hydrolysis while retaining the C1 configuration, the enzymes of GH19 and 48 utilize an inverting mechanism, while for one GH23 chitinase, a tunnel-like catalytic cavity relying on two acidic residues has been described [12]. GH18 chitinases utilize a conserved glutamic acid residue in the catalytic center and produce single GlcNAc units, disaccharides or higher oligosaccharides.

To date, the structure, function, and biotechnological application of primarily bacterial and fungal GH18 chitinases have been the focus of further studies [13]. However, research on the role of chitinases in human health has grown after the publication of the human genome. Humans express an acidic mammalian chitinase connected with asthma in addition to other chitinases or chitinase-like proteins of the family GH18 [14,15]. Recent studies have focused on proteins of human commensal organisms, such as intestinal bacteria [4,16], which can serve either as markers of health state, potential active substances against candidoses or triggers for colon-specific drug delivery [17].

*Clostridium paraputrificum* J4 is one of the bacterial species in the gastrointestinal tract of a healthy human. The strain *C. paraputrificum* J4 was discovered by Simunek et al. [4] as a strictly anaerobic bacterium, which can survive on chitin as the sole source of carbon. We have identified at least five different chitinases in the extracellular extracts of this bacterium [16] and have characterized the main components with endo-, exochitinase and *N*-acetylglucosaminidase activity. Complete genomes of thirty-two other clostridia, excluding *C. paraputrificum* J4, have been annotated (NCBI, http://www.ncbi.nlm.nih.gov/genome/, accessed on 25 April 2021), twenty of which do not contain any chitinase and only one that has at least two predicted chitinases. Within clostridia, only *C. paraputrificum* J4 harbors genes for such a high number of chitinolytic enzymes.

*C. paraputrificum* J4 secretes enzymes in support of its nutrition in the human intestine, where chitin or chitooligosaccharides cannot be normally processed by human enzymes, which raises questions about the source of chitin in humans. Chitin, chitosan, and derived substances have been used in the food industry as antimicrobial agents, for beverage clarification, and in pharmaceutical and therapeutic applications, etc. [18,19]. These applications, apart from food contamination by fungi, explain the occurrence of a commensal bacterium well equipped for chitin degradation. Fungal infections can become a major issue in human health and more effective means of treatment are sought [20]. Chitinases, capable of degradation of chitin-containing fungal cell walls and originating from human symbionts, may be used in applications against candidoses or in targeted drug release [21].

The research efforts directed at the understanding of the interactions of the SARS-CoV-2 virus within humans, indicated, among other effects, infection-related changes in microbiome composition [22] as an important direction for diagnostic development and understanding the fitness of the human immune system. Better understanding of the molecular details behind microbiome development and interdependencies between a healthy human and commensal species should provide a solid base for further developments in diagnostics and treatment of pathogen-borne diseases.

In this work, we characterized an isolated form and two recombinant forms of the most abundant chitinase of the human commensal bacterium *Clostridium paraputrificum* J4. We bring functional analysis and insights into the domain structure of the major chitinase of this bacterium and evidence of gene/enzyme adjustment to the typical conditions of the human colon environment.

## 2. Materials and Methods

### 2.1. Bacterium Cultivation and Culture Fluid Treatment

A strictly anaerobic mesophilic chitinolytic bacterial strain *C. paraputrificum* J4 was isolated from human feces [23]. The final identification was performed by sequencing of a 16S rDNA fragment (GenBank accession no: KX766027.1, UniProtKB accession no.: A0A1C9J7J5). The bacterium was grown on modified medium M10 [24], containing colloidal chitin (4 g/L) as the substrate to induce the synthesis of chitinolytic enzymes. At the exponential phase of growth, the cells were harvested by centrifugation at 35 000× *g* for 15 min at 4 °C.

### 2.2. Purification and Identification of Crude Enzyme crChit62J4

Sample preparation for preparative chromatographic separation of the chitinolytic enzymes from the supernatant of the culture fluid is described in [25]. Briefly, the bacteria-free supernatant was filtered through 100-kDa and 30-kDa PES (polyethersulfone) Millipore membranes under an applied pressure of 50 kPa to partially separate solutes with molecular weight (MW) higher than 100 kDa and lower than 30 kDa, respectively. The main fraction of chitinolytic enzymes was retained with a PES membrane with 30 kDa cut-off. It was approximately 25× concentrated.

The retentate was dialyzed (3 kDa cut-off, Spectrum Medical Diagnostics Inc., Mississauga, ON, Canada) against the buffer A (0.05 M Tris-HCl, pH 7.5) and loaded onto a pseudo-affinity support (strong anion-exchange resin DOWEX-1×2 with fixed counterions of EDTA). It showed strong affinity to colored admixtures accompanying the target enzymes. Several of the chitinolytic enzymes left the pseudo-affinity column at sorption and during washing with loading buffer A. Adsorbed chitinolytic enzymes were desorbed under a linear gradient of NaCl (0–0.5 M) in buffer A. The chitinolytic enzymes were eluted in two strongly overlapping peaks in the 0.02–0.1 M NaCl range. The combined fractions of the second and the third peak were dialyzed (3 kDa cut-off) against buffer A, concentrated by vacuum evaporation and purified on a DEAE Sepharose Fast Flow anion exchange column under the same sorption and elution conditions. 

Enzymes were further separated by size exclusion chromatography using HiLoad 16/60, Superdex 75 prep grade equilibrated with 50 mM HK_2_PO_4_/H_2_KPO_4_, 150 mM NaCl, pH 7.0 and then polished by chromatofocusing using MonoP 5/200 GL equilibrated in 0.25 M BisTris, pH 7.5 and eluted by Polybuffer74, pH 4.0 (all columns by GE Healthcare Life Sciences, Marlborough, MA, USA). The enzyme was concentrated in 0.1 M HK_2_PO_4_/H_2_KPO_4_, pH 7.0 to 4.5 mg/mL. Sample purity was verified using sodium dodecyl sulfate (SDS) gel electrophoresis and activity using zymograms [4]. Molecular mass analysis was performed on a subset of enzymes using matrix-assisted laser desorption/ionization-time of flight mass spectrometry (BIFLEX III spectrometer, Bruker). Preselected samples according to mass estimates and detected activity were submitted for peptide fragment mass spectrometry analysis.

### 2.3. Recombinant Expression

DNA coding for both rChit62J4 (resulting product sequence, tag and cleavage site residues are underlined: MGSSHHHHHHSSGENLYFQGGTHMLEAQSL…WQKQ, 598 amino acid residues, theoretical MW 65.222 kDa) and rChit62J4cat (MGSSHHHHHHSSGENLYFQGGTHMLEAQSL…LTPV, 357 a.a., 39.395 kDa) was amplified by PCR using primers incorporating *XhoI* and *BamHI* restriction sites (Supplementary Table S1) and subcloned into a pET-15bTEV expression vector. The DNA sequences were verified by sequencing using vector-specific primers. Proteins were heterologously expressed in *E. coli* BL-21 (DE3) in LB medium induced by the addition of 0.5 mM IPTG (isopropyl β-d-1-thiogalactopyranoside) at 37 °C. Cells were disrupted using sonication after 4 h of target protein expression. The proteins were purified using a Ni-NTA Superflow™ gravity column (QIAGEN, Hilden, Germany), washed with 50 mM imidazole and eluted with 200 mM imidazole. The last step in protein purification was size exclusion chromatography using Superdex 200 (buffer 0.5 M H_2_KPO_4_/HK_2_PO_4_, pH 7), and isofocusing using a MonoP column equilibrated in 0.025 mM BisTris, pH 7.0 pH and eluted with a pH gradient by Polybuffer75 pH 4.0. The quality of the resulting protein samples was monitored using SDS-PAGE (Supplementary Figure S1).

### 2.4. DNA Isolation and Genomic Sequencing

DNA of *C. paraputrificum* J4 was isolated from 3-days-old culture using the QIAamp DNA Stool Mini kit (QIAGEN, Hilden, Germany). Shotgun genomic sequencing using Illumina Hiseq2000, together with genome assembly and initial bioinformatics analysis was performed by CD Genomics (Shirley, NY, USA). Protein coding sequences contained the sequence of Chit62J4 together with other chitin-degrading enzymes.

### 2.5. Proteolytic Digestion and Mass Spectrometry Analysis

Crude enzyme was analyzed in protein bands cut from SDS denaturing gel and zymogram and in purified form using trypsin proteolysis and matrix-assisted laser desorption/ionization-Fourier transform mass spectrometry analysis. Protein bands were cut from gel, cut into small pieces, and decolorized in sonic bath at 60 °C several times with 0.1 M 4-ethylmorpholine acetate (pH 8.1) in 50% acetonitrile (ACN). After complete destaining, proteins were reduced by 50 mM tris-(2-Carboxyethyl)phosphine in 0.1 M 4-ethylmorpholine acetate (pH 8.1) for 5 min at 80 °C and alkylated using 50 mM iodoacetamide in 0.1 M 4-ethylmorpholine acetate (pH 8.1) for 30 min in the dark at room temperature. Then, the gel was washed with water, shrunk by dehydration with ACN, and reswollen in water. The rehydration and dehydration of the gel was repeated twice. Next, the gel was reswollen in 0.05 M 4-ethylmorpholine acetate (pH 8.1) in 50% ACN and then the gel was partly dried using a SpeedVac concentrator (Savant, Holbrook, NY, USA). Finally, the gel was reconstituted with cleavage buffer containing 0.01% 2-mercaptoethanol, 0.05 M 4-ethylmorpholine acetate (pH 8.1), 10% ACN, and sequencing grade trypsin (Promega, 10 ng/μL). Digestion was carried out overnight at 37 °C, the resulting peptides were extracted with 30% ACN/0.1% trifluoroacetic acid (TFA) and subjected to mass spectrometric analysis.

Mass spectra were acquired in the positive ion mode on a MALDI-FTMS APEX-Ultra (Bruker Daltonics, Bremen, Germany) equipped with a 9.4 T superconducting magnet and a SmartBeam laser. The acquisition mass range was 700–3500 *m*/*z* and 512k data points were collected. A 280 V potential was applied to the MALDI plate. The cell was opened for 2500 ms, 4 experiments were collected for one spectrum, where one experiment corresponds to 300 laser shots. The instrument was externally calibrated using PepMix II peptide standard (Bruker Daltonics, Bremen, Germany), resulting in a typical mass accuracy below 2 ppm. A saturated solution of α-cyano-4-hydroxy-cinnamic acid in 50% ACN/0.2% TFA was used as a MALDI matrix. 1 μL of matrix solution was mixed with 1 μL of the sample on the target and the droplet was allowed to dry at ambient temperature. After analysis, the spectra were apodized using square sin apodization with one zero fill. The interpretation of mass spectra was performed using DataAnalysis version 3.4 and BioTools 3.2 software packages (Bruker Daltonics, Billerica, MA, USA). Proteins were identified by peptide mass fingerprinting (PMF) using the search algorithm MASCOT (Matrix Science, Boston, MA, USA).

The characteristic spectrum was matched to the complete genome of *C. paraputrificum* J4. For purified crChit62J4 the characteristic spectrum was matched reliably with predicted fragments of putative chitinase from the bacterial genome (peg1890, Supplementary Figure S2).

### 2.6. MALDI-TOF Analysis

The crude enzyme crChit62J4 in 0.05 M Tris/HCl, pH 8, at a concentration of 1 mg/mL was subjected to matrix-assisted laser desorption/ionization-time of flight (MALDI-TOF) mass spectroscopy analysis (Supplementary Figure S3) with a BIFLEX III spectrometer (Bruker).

### 2.7. Activity Assay

Substrate specificity was determined using substrates 4-nitrophenyl-*N*-acetyl-β-d-glucosaminide (pNG), 4-nitrophenyl-*N*,*N*′-diacetyl-β-d-chitobioside (pNGG), and 4-nitrophenyl-β-d-*N*,*N*′,*N*′′-triacetylchitotrioside (pNGGG). The reaction mixture with a total volume of 80 μL containing pure enzyme at concentration 2.7 μg/mL, substrate at concentration 2 mM, and Assay buffer (60 μL, Sigma–Aldrich, St Louis, MO, USA, A4855) was incubated at 37 °C and the reaction was stopped by the addition of 0.4 M Na_2_CO_3_ (40 μL) after 45 min. Increase of optical density at 405 nm was measured with respect to background readings for identical mixtures without enzyme. 

The effects of pH on activity, optimum temperature and kinetic parameters were measured using pNGG and standard assay conditions. The pH optimum was found using 0.1 M citric acid–potassium phosphate buffer (pH 3–7), 0.1 M potassium phosphate buffer (pH 6–8) and 0.1 M glycine buffer (pH 9–10). The temperature optimum was determined in the range of 40–90 °C in 0.1 M citric acid–potassium phosphate, pH 5.5.

Kinetic parameters were calculated from initial rate parameters with concentrations of pNGG from 0.02 to 4.2 mM in 0.1 M citric acid–potassium phosphate buffer pH 5.5, and incubation time 30 min (linear increase was observed in the first 70 min of reaction). *K*_m_ and *k*_cat_ were calculated from an average of three measurements according to the Michaelis-Menten equation. The kinetics interpretation and fitting were performed using GraphPad Prism version 7.02 for Windows (GraphPad Software, La Jolla California USA, www.graphpad.com). Activity on colloidal chitin was checked using 0.5% (*w*/*v*) carboxymethyl chitin as a substrate according to Inglis and Peberdy [26].

The influence of selected compounds on the activity and kinetic parameters were investigated. The protein sample was pre-incubated with each reagent in the reaction buffer at room temperature for 20 min. Then, it was used to measure the change in activity and Michaelis-Menten dependence as described above. The final concentration of the reagent in the reaction was 5 mM.

### 2.8. TLC

Thin-layer chromatography (TLC) was used to analyze the reaction products with chitin, chitohexaose, and CM-chitin as substrates. Aliquots of the reaction mixtures were chromatographed on a silicagel sheet (ALUGRAM SIL G Art.Nr. 818163, Thermo Fisher Scientific, Waltham, MA, USA) with *n*-butanol–methanol–25% ammonia solution–water (volume ratio 5:4:2:1). The products were developed using a spray containing aniline–diphenylamine reagent (4 mL of aniline, 4 g of diphenylamine, 200 mL of acetone, and 30 mL of phosphoric acid) and baking the sheet at 180 °C for 3 min [27]. For the raw TLC data see the photographs of the TLC plates (Supplementary Figure S4).

### 2.9. Dynamic Light Scattering

Particle size distribution was assessed using dynamic light scattering (DLS, Malvern Instruments, ZEN3600) in a 45-μL glass cuvette at 18 °C with enzyme concentration 1.0 mg/mL in 50 mM KH_2_PO_4_/K_2_HPO_4_, 1 mM NaN_3_, pH 7.0. MW was estimated using the empirical mass vs. size calibration curve (Dispersion Technology Software 5.03, Malvern Instruments).

### 2.10. Determination of Protein Concentration

Protein concentration was estimated based on UV spectrophotometry at λ = 280 nm in 1 cm path-length quartz cuvette, using a Libra 22 UV-VIS spectrophotometer (Biochrom, United Kingdom) with background reading for a given buffer and a theoretical extinction coefficient based on the protein sequence.

### 2.11. Sequence Analysis and Computer Modeling

Sequence searches were performed using the BLAST service [28,29]. Sequence alignments were performed with ClustalX and ClustalW using the Gonnet 250 weight matrix [30]. Enzyme domains were modeled using the SwissModel server, ProMod3 3.2.0 [31]. Models were calculated based on automated or manual lead selection and preselected homologous domains identified by sequence search. Models with an overall QMEAN4 global score greater than 0.6 were accepted [32]. 

## 3. Results

**Figure 1:**
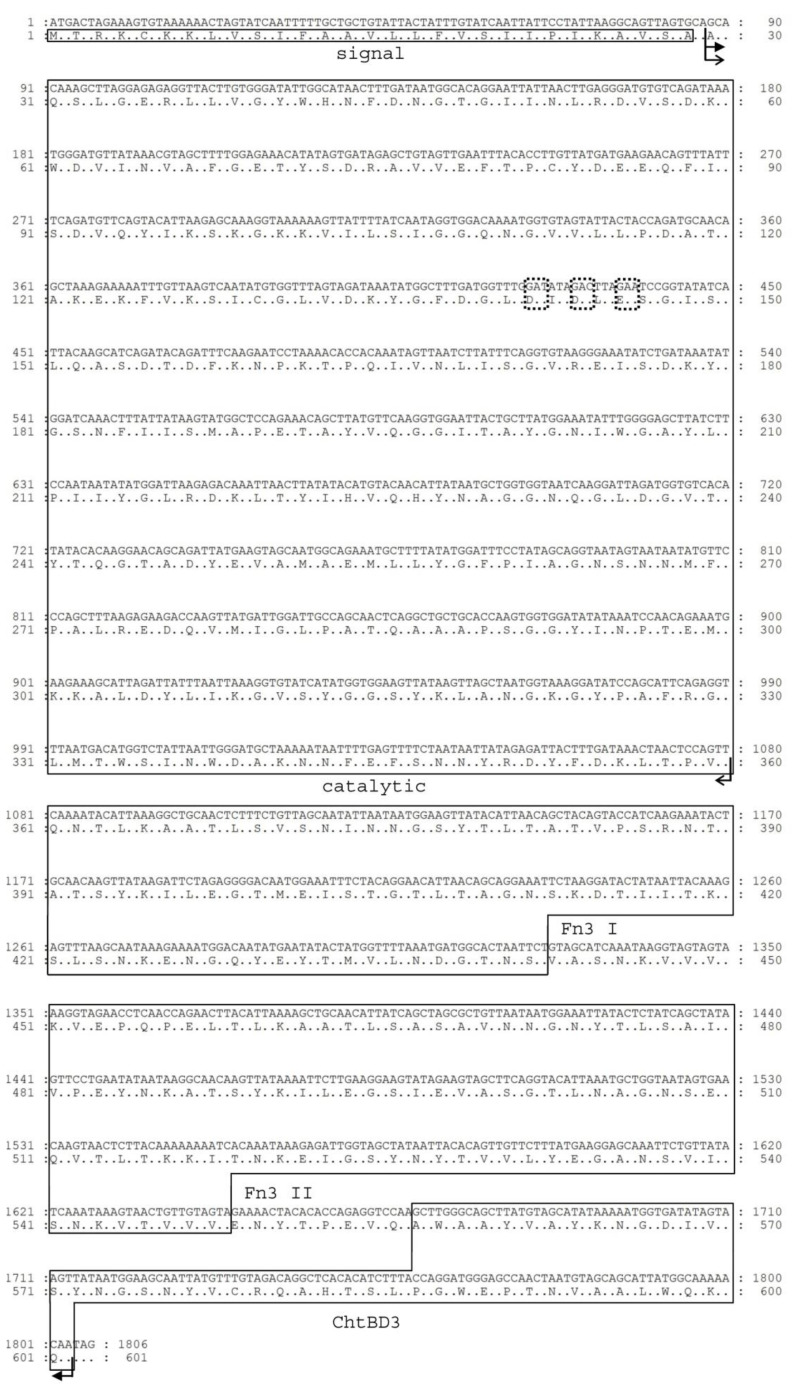
DNA and amino acid sequence of chitinase Chit62J4. The expected domain boundaries and catalytic amino acids are marked. The construct boundaries of rChit62J4 and rChit62J4cat are marked with filled and line arrows, respectively.

Anaerobic cultivation yielded approximately 0.5 mg of crChit62J4 per liter of culture, after purification providing ~ 0.1 mg of protein per liter of initial culture volume. The identity and purity of crChit62J4 were confirmed by MS analysis and reliable sequence match. The Chit62J4 gene codes for 601 amino acids with a total theoretical MW = 65414.9 and pI = 5.81 (Figure 1, Supplementary Figure S5). This includes a signal sequence identified by the prediction services as residues 1–29, i.e., the *N*-terminus of the mature enzyme sequence being AQSL [33,34].

The theoretical mass of mature Chit62J4 (residues 30–601) of 62284.9 and pI of 5.2 correspond to the experimental values for crChit62J4: 62.4 kDa (MALDI-TOF, Supplementary Figure S3) and pI of 4.9 (chromatofocusing). The DLS results for crChit62J4 indicate a monodisperse solution with hydrodynamic radius 3.65 nm (~ 130 kDa for globular protein) corresponding to a dimer of Chit62J4. If Chit62J4 had an extremely elongated shape, it may also roughly correspond to a monomer of the enzyme.

Crude Chit62J4 is active on pNGG and pNGGG (against pNGGG ~10× lower, data not shown), with no activity toward pNG. On these chromogenic substrates, the enzyme performs mostly as an exochitinase, on pNGG exhibiting *k*_cat_ 13.5 s^−1^, *K*_m_ = 0.57 mM and catalytic efficiency *k*_cat_/*K*_m_ = 23,684 M^−1^s^−1^, optimal pH 5.5 and temperature 60 °C (Supplementary Figure S6). The enzyme is also active on colloidal chitin. In the presence of standard antimycotics Griseofulvin, Amfotericine, and Clotrimazole (5 mM) it retains its full activity.

The predicted membrane translocation signal sequence at the *N*-terminus of the native Chit62J4 is followed by an amino acid sequence corresponding to a four-domain protein. Based on the sequence alignment with other known chitinases (Supplementary Figure S7), we propose the following domain structure of mature Chit62J4: the N-terminal catalytic domain, two re-iterated Fn3/Big3 domains, and the C-terminal chitin binding module ChtBD3 (chitin-binding domain type 3). The overall structure resembles that of chitinase Chi18C from *Clostridium paraputrificum* M21 [35].

The kinetic parameters for both rChit62J4 and rChit62J4cat toward pNGG were determined as *K_m_* = 0.24 ± 0.02 mM, *k*_cat_ = 31.5 ± 0.9 s^−1^ and *K_m_* = 0.19 ± 0.02 mM, *k*_cat_ = 31.4 ± 0.8 s^−1^, respectively. The influence of several groups of compounds expected to affect activity was tested. In the case of the catalytic domain, the substrate affinity increased slightly compared to the complete mature enzyme. The affinity also increased in the presence of 5 mM MgCl_2_, 5 mM EDTA, 5 mM ZnSO_4_, and 5 mM CaCl_2_ (Table 1; Supplementary Figures S8 and S9). Conversely, the substrate affinity decreased in the presence of 5 mM glucosamine or 5 mM glucose. The TLC results showed that both the intact enzyme and its catalytic domain cleaved chitohexaose as well as colloidal chitin producing chitobiose as the end product (Supplementary Figure S4).

## 4. Discussion

### 4.1. Sequence and Structure of Chit62J4

The most abundant extracellular enzyme of *Clostridium paraputrificum* J4 is chitinase Chit62J4 with predominant exochitinolytic activity and belonging to the GH18 family with the retaining mechanism. Mature Chit62J4 comprises four domains: the catalytic domain, two copies of a Fn3 domain, and the carbohydrate-binding module ChtBD3 (chitin-binding domain 3). This domain structure is not uncommon in chitinases, the most similar enzyme being Chi18C from *Clostridium paraputrificum* M21, however, with different catalytic domain and specificity. A sequence search against known structures in the Protein Data Bank does not provide significant hits.

The catalytic domain of Chit62J4 shares the highest sequence identity (79%) with single domain chitinase D from *Clostridium botulinum* B str. Eklund 17B (NCBI YP_001885576.1). Of the chitinases with known 3D structures, the catalytic domain of Chit62J4 shows the highest similarity (75% identity) to the single domain chitinase ChiNCTU2 from *Bacillus cereus* NCTU2 [36]. Thus, while the complete domain structure of Chit62J4 most resembles that of Chi18C, the catalytic properties should resemble those of ChiNCTU2. The explanation for this “catalytic domain replacement” remains elusive. The accessory domains likely contribute to better binding and directionality of the substrate with respect to the catalytic site and support catalysis on fibers.

The 90-residue Fn3/Big3 domains (residues 363–442 and 449–548) share 63% identity and show 44–57% identity to the closest homologous sequences in chitinases, e.g., in Chi18C. Pfam [37] classifies the first Fn3 domain into the Cadherin family or family DUF4397 (“domain of unknown function in bacteria, archaea, and eukaryotes”) and the second domain as part of the CARDB family (“cell adhesion domain found in bacteria”). In our general sequence search against all protein records, the first Fn3 domain is most similar to two domains from *Clostridium beijerinckii* NCIMB 8052 carbohydrate-binding family V/XII protein (residues 229–317, 58% identity and 135–222, 49%). The second domain search leads to similar results. The complete two-domain sequence does not produce any significant hits, making the Fn3-Fn3 combination of domains in Chit62J4 unique.

The C-terminal domain ChtBD3 (557–601) is 60% identical with a carbohydrate-binding module of chitinase A1 from *Bacillus circulans* [38], a domain found by Hashimoto et al. [39] to enhance the enzymatic activity by interaction with insoluble chitin, while its interaction with soluble chitin or chitin derivatives was not observed.

### 4.2. Molecular and Catalytic Properties of Chit62J4

The isolated enzyme is present in solution in the form of monomers or dimers and its highest activity against pNGG compared to longer and shorter substrates characterizes it as a chitinase with predominant exochitinolytic activity. Its *K*_m_ and *k*_cat_ values are comparable to those of other chitinases (Table 2). The enzyme is active in pH range 3.5–7 with an optimum at pH 5.5. The optimal activity temperature of 60 °C indicates a highly stable enzyme, compared to other chitinases of similar sequence and structure. The absence of the non-catalytic domains in Chit62J4cat shifted the pH optimum to more acidic 5.0 (Supplementary Figure S6).

**Figure 2:**
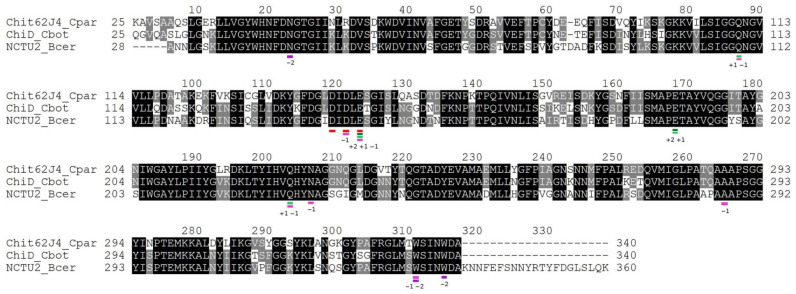
Sequence alignment of Chit62J4 from *C. paraputrificum* J4, chitinase D from *C. botulinum* and chitinase ChiNCTU2 from *B. cereus*. Identities are marked by black background. Expected catalytic residues are marked by a red line; residues forming the −2 to +2 subsites (according to Hsieh et al. [36]) of the substrate binding site are marked by colored lines and site numbering.

The key catalytic residues and the nearest surrounding amino acids of Chit62J4, ChiD and ChiNCTU2 are identical (Figure 2), which suggests comparable parameters for the catalytic mechanism and kinetics. The catalytic rates of rChit62J4 and ChiNCTU2 toward pNGG were 31.5 and 20.9 s^−1^, respectively, which are comparable. The *K*_m_ values of 240 μM (rChit62J4) and 74 μM (ChiNCTU2) imply the affinity of Chit62J4 to the substrate being approximately 3× weaker and the catalytic efficiency about 4× lower. The level of sequence identity enables reliable modeling of the catalytic domain of Chit62J4. Differences between ChiNCTU2 and Chit62J4 active sites can only be found further away from the catalytic center, certainly out of the reach of substrate subunits −2 to +2 (comparison of the model with superimposed structures with PDB id 1e6r, 1e6n, 1e6z, [44]; 1ehn, [45] 3n18, 3n12, [36]). Therefore, the different *K*_m_ values can be attributed most likely to the dynamic behavior and the overall differences in the electrostatics of the domain. ChiNCTU2 adjusts the conformation of the loop 85–88 at substrate binding. This requires a degree of dynamic flexibility, possibly different in ChiNCTU2 and Chit62J4. The accessory domains of Chit62J4 most likely do not contribute to the difference in the catalytic efficiency, and they do not affect the catalytic domain functionality, as their removal did not lead to significant changes of *K*_m_ or *k*_cat_. It is still expected, however, that the ChtBD3 domain would help to recruit and bind longer chitin or chitin-like substrates and increase the probability of contact with the Chit62J4 active site.

Based on the high sequence similarity and similar kinetics, we conclude that Chit62J4 utilizes the retaining mechanism with the catalytic residues Asp142, Asp144 and Glu146 and residues Tyr41, Phe68, and Gln110, conserved with ChiNCTU2, being the key residues for substrate binding [44]. This corresponds to the substrate-assisted catalytic mechanism of GH18 chitinases via bicyclic oxazolinium-ion intermediate [11].

### 4.3. Adaptation of Clostridium Chitinase to Environment

Chitinases of this family have optimal temperatures in a wide range of 30–60 °C and optimal pH mostly in the range 5.0–8.0 with several examples of extremes at pH 4.0 and 9.0 [35,46]. Within related enzymes, there is no example of a chitinase with such a high-temperature stability and at the same time such a low pH optimum. The catalytic domains of ChiNCTU2 and Chit62J4 are highly similar and according to our structure model (see below) the core of the catalytic site should be almost identical. Their temperature optima are equal, however, the Chit62J4 pH optimum of 5.5 contrasts with that of ChiNCTU2 at pH 7.0. This significant difference is, nevertheless, in agreement with the surface electrostatics (Supplementary Figure S10) and the theoretical isoelectric points of their catalytic domains. More acidic Chit62J4 (negative surface potential, pI 4.9) has a lower pH optimum than ChiNCTU2 with pI 6.0. The theoretical and experimental (native conditions) pI of complete mature Chit62J4 are 5.2 and 4.9, respectively. In both enzymes, the pH optimum is several tenths to one degree above the respective pI. Given the high identity of the active sites, the difference in pH optima between ChiNCTU2 and Chit62J4 results rather from the overall protein behavior at a certain pH than from any particular variations in the active site. Our calculations of electrostatics distributions at the measured pH optima show for both ChiNCTU2 and Chit62J4 typical chitinase patterns (Supplementary Figure S10), with no significant positive potential and relatively high negative potential at the active center. Chitin chains, often partially deacetylated, can carry positive charge and positive potential near the active site would block substrate binding, while strong negative potential will lead to nonproductive binding. Calculations performed at the exchanged pH points for the two proteins clearly show extreme electrostatics (Supplementary Figure S10). Therefore, the optimization with respect to the acidity of the environment in these enzymes was achieved by adjusting the overall molecular electrostatics, coded by the amino acid composition (different) and three-dimensional organization (similar) without any interference directly in the active site. ChiNCTU2 originates from a soil bacterium, while Chit62J4 is active in the human colon. pH within a healthy human colon slowly increases from 5.5 at the beginning to 7.0 at its end [47]. The *Roseburia*/*E. rectale* group of bacteria in the study by Duncan et al. (2009) [6] competed well at lower pH for polysaccharide substrates. It follows from this that if *Clostridium* needs to compete for insoluble fiber substrates, it must compete at decreased pH in the proximal part of the colon. Thus, the optimization for chitinolytic activity at lower pH is in full agreement with the microbiome environmental conditions. The highest bacterial growth occurs in the first colon section, and it can be expected that *C. paraputrificum* J4 is exposed to lower pH than *B. cereus*, under competition with other microbiome bacteria. Adjustment of extracellular Chit62J4 to the acidic environment of the human colon therefore explains the differences observed on the molecular level. The Chit62J4 adaptation happened away from the actual catalytic site. Similar shifts of pH optima, realized by the overall protein composition rather than active site changes, were seen also in other enzymatic systems, e.g., in non-specific nucleases [48].

Utilization of carbohydrate-binding domains by commensal bacteria enzymes represents an important strategy for providing the ability to process fibrous chitin and for localization closer to the energy source, both as advantages in internal microbiome competition [1]. Chit62J4 is capable of CM-chitin degradation to chitobiose, both in its intact form rChit62J4 and as the sole catalytic domain rChit62J4cat. Chit62J4 is thus capable of adsorbing on the surface of chitin particles and processing the accessible ends of chitin fibers.

The existence of a chitinase-coding gene, which has a catalytic domain remarkably similar to a typical soil bacterium with shifted pH optimum and complemented by accessory domains, illustrates the adjustments needed to utilize chitin and chitin-derived compounds in the human colon. Microbiota adaptation to diet was previously also proven on the level of enzyme functionality [49]. Similarly, *Clostridium paraputrificum* J4 appears to have its cohort of enzymes adjusted to the gut environment.

### 4.4. Role of Chit62J4

*C. paraputrificum* J4 employs several different enzymes in the individual steps of degradation of chitin [17]. Endochitinases perform the initial steps to produce the free ends of chitin chains. Exochitinases, including Chit62J4, then process chains with free ends (possibly still attached to the fibrous substrate) into chitobiose units. N-acetylglucosaminidases produce individual GlcNAc units, which are then most likely imported and utilized by *C. paraputrificum* J4, mainly as an energy source. Under varied cultivation conditions, Chit62J4 remains at high levels and the highest of the 60 kDa isoenzymes without dependence on cultivation parameters, which underlines its importance in the chitinolytic complex (Chit62J4 most likely corresponds to samples “III” and “C27” in previously reported isolation experiments by Dušková et al. [16] and Šimůnek et al. [23], respectively).

### 4.5. Factors Influencing Activity of Chit62J4

Mercury and iron are almost universal enzyme inhibitors and in the case of crChit62J4 3% and 58% of activity is retained, respectively (Table 3). Inhibition is observed with Mn^2+^ and Cu^2+^. Ca^2+^ has no effect, in contrast to a 100% increase of the activity of ChiNCTU2 [43]. Mg^2+^ causes a small increase in affinity, which was also observed for chitinase CHIT60 from *Serratia plymuthica* HR0-C48 [50] and a 60 kDa chitinase from *Bacillus* sp.13.26 [51]; ChiNCTU2 was not affected (Table 4). The exchanged effects of Ca^2+^ and Mg^2+^ on the two highly similar active sites (Chit62J4 vs. ChiNCTU2) can be explained only by differences in the overall composition/structure and not by direct effects on the active site as the direct participation of metals in the mechanism can be excluded (no effect of 5 mM EDTA) and the active centers are identical. Binding of Mg^2+^ was reported in two chitinase structures [52], however, without any explanation of the role in catalysis. 

### 4.6. Potential Application with Antimycotics

As suggested by Davies and Pope [58], chitinases can be applied with low molecular weight antimycotics. Cell walls of pathogens causing mycoses, such as *Candida albicans* and dermatophytes *Microspora*, *Trichophyton*, and *Epidermophyton* [59,60,61] include chitin and often also mannan. Enzymatically damaged walls would be more easily penetrated by standard antimycotics, which will increase the efficiency of treatment. For such applications, the formulation of a combination of non-interfering enzymes with standard drugs would be necessary. Chit62J4 is a suitable candidate as antimycotics do not influence its activity.

*Clostridium paraputrificum* J4 secretes its main GH18 chitinase Chit62J4, relying on a catalytic domain optimized for low pH and accessory domains important for fibrous substrate degradation. Chitinases with highly homologous catalytic domains show significantly different parameters of catalysis (ChiNCTU2 and Chit62J4). Thus, catalytic properties cannot be reduced to the properties of the catalytic domains and of the active site but depend on the build-up of the whole protein and possibly on the dynamics of enzyme-substrate interactions as well. The results show the potential for fine-tuning of the catalytic properties of a chitinase for biotechnological applications as well as for tailored drug delivery systems relying on chitinolytic activity in the gastrointestinal tract. For potential applications in antifungal cocktails, standard antimycotics would not interfere with the catalytic activity of Chit62J4, which can be targeted against the chitin components in the fungal cell wall.

## Figures and Tables

**Table 1:** Kinetic parameters of rChit62J4 influenced by selected compounds. Standard deviations are given.

Enzyme	Compound Added into the Reaction Trial	*k*_cat_ (s^−1^)	*K*_m_ (mM)
rChit62J4	none	31.5 ± 0.9	0.24 ± 0.02
rChit62J4	5 mM glucosamine	30.2 ± 0.9	0.36 ± 0.04
	5 mM glucose	31.2 ± 0.9	0.36 ± 0.04
	5 mM MgCl_2_	29.6 ± 0.6	0.18 ± 0.02
	5 mM EDTA	31.9 ± 0.8	0.19 ± 0.02
	5 mM ZnSO_4_	31.1 ± 0.6	0.16 ± 0.01
	5 mM CaCl_2_	30.3 ± 0.6	0.14 ± 0.01
rChit62J4cat	none	31.4 ± 0.8	0.19 ± 0.02

**Table 2:** Comparison of activity of chitinolytic enzymes related to Chit62J4.

Enzyme	Organism	*M*_r_ kDa	Stability Range pH	Optimal pH, Temperature	Substrate Type and Assay Conditions	*K*_m_ (μM)	*V*_max_ (μmol/min/mg)	Activity Type	Inhibitors	Reference
Chitinase A	*Clostridium paraputrificum* M-21	89.0	6–9	6, 45 °C	pNGG, pH 6, 37 °C	6.9	43.0	Exo	1 mM HgCl_2_ Partly: AlCl_3_, CaCl_2_, CuCl_2_, FeCl_3_, MnCl_2_ Enhanced: MgCl_2_	Morimoto et al. [40]
Chitinase B	*Clostridium paraputrificum* M-21	86.5	6–9	6, 45 °C	pNGG, pH 7, 37 °C	6.3	46.0	Exo	1 mM HgCl_2_ Partly: AlCl_3_, CuCl_2_, FeCl_3_, No Effect: MgCl_2_, CaCl_2_, EDTA	Morimoto et al. [41]
Chitinase C	*Clostridium paraputrificum* M-21	72.0	NA	6, 60 °C	pNGGG, pH 6, 50 °C	0.44	26.6	Endo		Morimoto et al. [35]
*N*-acetylglucosaminidase	*Clostridium paraputrificum* M-21	45.5	6–9	7, 50 °C	pNG, pH 7, 37 °C	7.9	21.8	NAGase		Li et al. [42]
rChit62J4	*Clostridium paraputrificum* J4	62.3		5.5, 60 °C	pNGG, pH 5.5, 37 °C	240	29.0	Exo	5 mM MnSO_4_, ZnSO_4_, FeCl_2_, HgCl_2_	This work
ChiNCTU2	*Bacillus cereus* NCTU2	36.2	6–8	7.0, 50–60 °C	pNGG, pH 6.5, 25 °C	74	34.6	Exo	10 mM Hg^2+^, Cu^2+^, Zn^2+^	Hsieh et al., Wen et al., [36,43]

**Table 3:** Inhibition data for crChit62J4. Measured with 2 mM pNGG as a substrate, 2.7 μg/mL crChit62J4, at 37 °C and pH 5.5. The relative values of activity compared to values without added compounds under the same conditions and standard deviations are given.

Inhibitor	% Activity		
5 mM MnSO_4_·H_2_O	52	±	6
5 mM CuSO_4_·5H_2_O	89	±	10
5 mM FeCl_2_·4H_2_O	58	±	9
5 mM HgCl_2_	3	±	4
5 mM Griseofulvin	104	±	4
5 mM Amfotericine	119	±	27
5 mM Clotrimazole	102	±	4

**Table 4:** Inhibition and stimulation effects in selected chitinases. Inhibitor concentration was 5 mM unless a different value is given in brackets. Percentage of original activity is given. Values marked with * are the mean values for substrate concentration 3.5 mM from kinetics in this work.

Organism, Reference	*Serratia plymuthica* HR0-C48 [50]	*Serratia plymuthica* HR0-C48 [50]	*Scorpaena scrofa* [53]	*Bacillus cereus* NCTU2 [43]	*Enterobacter* sp. G-1 [54]	*Alcaligenes xylosoxydans* [55]	*Bacillus* sp. 13.26 [51]	*Bacillus* sp. BG-11 [56]	*Clostridium* sp. JM2 [57]	*Clostridium* sp. JM2 [57]	This Work
Enzyme, MW (kDa)	Chit60, 60	Chit100, 100	Chit50, 50	ChiNCTU2, 36	ChiA, 60	Chitinase, 45	Chitinase, 60	Chitinase, not given	Purified chitinolytic complex	Purified chitinolytic complex	Chit62J4, 62
Substrate, (concentration if known, mM)	pNGG (10)	pNG (10)	Chitin (5)	Chitin (10)	Chitin (10)	Chitin	Chitin	Chitin	pNG (10)	Colloidal chitin (10)	pNGG (2)
Mn^2+^	250	55	145	100			0		10	10	52
Ca^2+^	120	50	66	200	105	100	50	120			97 *
Cu^2+^	10	0	103	5		25			20	12	89
Mg^2+^	115	90	61	100		100	131		110	77	89 *
Hg^2+^			0	5			0	50	0	0	3
Co^2+^	150	5	325					50			
Zn^2+^			111						10	27	98 *
Ba^2+^			90	100		100					
Cr^2+^				100							
Fe			85						0	0	58
K^+^			128						100	75	
Ag^+^			15					50	14	6	
Na^+^	100 < 0.5 M>	100 < 0.5 M>			100	25					
Ni^2+^							116	120			
EDTA					58			50	0	40	97 *

## Data Availability

The data presented in this study are available on request from the corresponding author.

## Supplementary Materials

Electronic supplementary material is available: Figure S1: SDS-PAGE of the reported proteins. Figure S2: Peptide mass fingerprinting data for crChit62J4. Figure S3: MALDI-TOF spectrum of crChit62J4. Figure S4: TLC raw data. Figure S5: Sequence records of Chit62J4. Figure S6: Graphs of pH and temperature dependence of crChit62J4 activity and pH dependence of rChit62J4cat activity. Figure S7: Proposed domain structure of Chit62J4. Figure S8: Michaelis-Menten kinetics curves for Chit62J4 in the presence of selected compounds. Figure S9: Overlay of Chit62J4 kinetics in the presence of selected compounds. Figure S10: Electrostatic surface potential of the catalytic domain of Chit62J4 and chitinase ChiNCTU2. Table S1: Oligonucleotides applied for PCR amplification of rChit62J4 and rChit62J4cat.

## Sample Availability

Plasmids used for recombinant production of rChit62J4 and rChit62J4cat are available from the authors.

## Abbreviation

CBM	carbohydrate binding module
EDTA	ethylendiaminetetraacetic acid
GH	glycosyl hydrolase
GlcNAc	N-acetylglucosamine
PES	polyethersulfone
SDS	sodium dodecyl sulfate
MALDI-TOF	Matrix-assisted laser desorption/ionization-time of flight
CAN	acetonitrile
TFA	trifluoroacetic acid
MALDI-FTMS	Matrix-assisted laser desorption/ionization-Fourier transform mass spectrometry
PMF	peptide mass fingerprinting
CM-chitin	carboxymethyl chitin
MS	mass spectrometry
DLS	Dynamic light scattering
Fn3	Fibronectin type 3
ChtBD3	chitin-binding domain type 3
PDB	Protein Data Bank
pNG	4-nitrophenyl-N-acetyl-β-D-glucosaminide
pNGG	4-nitrophenyl N
pNGGG	4-nitrophenyl-β-d-*N*,*N*′,*N*′′-triacetylchitotrioside
The nucleotide and amino acid sequences of Chit62J4 are available in the GenBank database under accession code KX353699.	
