# Supplementary material for: Chitinase Chit62J4 Essential for Chitin Processing by Human Microbiome Bacterium Clostridium paraputrificum J4
Source: Molecules. 2021 Oct 2;26(19):5978. doi: 10.3390/molecules26195978 (PMC8512545; doi:10.3390/molecules26195978)
Supplement: Supplementary file 1 [file molecules-26-05978-s001.zip › molecules-1304922-supplementary.pdf]

Supplementary data for

Chitinase Chit62J4 essential for chitin processing by human microbiome bacterium *Clostridium paraputrificum* J4

by
Jan Dohnálek, Jarmila Dušková, Galina Tishchenko, Petr Kolenko, Tereza Skálová, Petr Novák, Karla Fejfarová, and Jiří Šimůnek

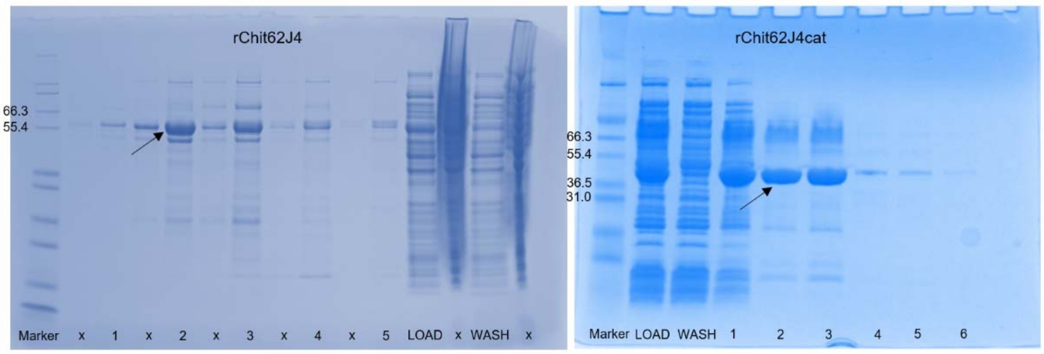

Supplementary Figure S1. SDS-PAGE analysis of purified rChit62J4 and rChit62J4cat by Ni-NTA affinity chromatography. The black arrow marks the target recombinant protein as verified by mass spectrometry peptide mapping. Left panel - rChit62J4 – lane content: Marker: molecular weight marker Mark 12^{TM} Unstained Standard from Novex, Life Technologies, 1-5: elution fractions of purified protein, load volume 5 µL, LOAD: culture lysate, 1 µL, WASH: column flow-through, 1 µL. Lanes marked x are the same fractions with a lower amount of loaded sample (for lanes 1-5, 1 µL) or a higher amount (lanes LOAD and WASH, 5 µL). Right panel - rChit62J4cat – lane content: Marker: molecular weight marker Mark 12^{TM} Unstained Standard from Novex, Life Technologies, LOAD: culture lysate, WASH: column flow-through, 1-6: elution fractions of purified protein.

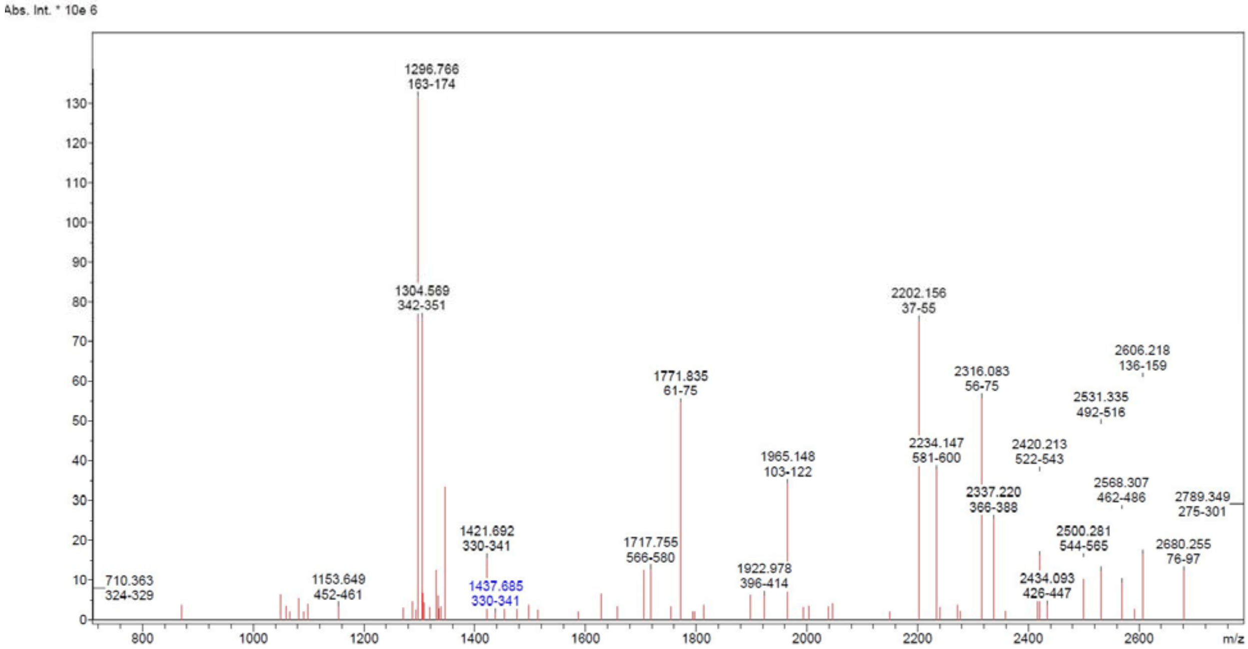

Peaklist:

Peak	Mass	Intensity	Peak	Mass	Intensity	Peak	Mass	Intensity	Peak	Mass	Intensity
1	710.363	4220078.000	2	871.029	3712087.000	3	1049.522	6406552.000	4	1060.064	3536853.000
5	1065.517	2029604.000	6	1082.054	5399797.000	7	1091.533	1953959.000	8	1098.028	3877401.000
9	1153.649	4507695.000	10	1271.088	3026842.000	11	1287.063	4601382.000	12	1293.079	2391077.000
13	1296.766	138514704.000	14	1304.569	77244144.000	15	1305.553	6738322.000	16	1309.054	4227188.000
17	1318.748	3179130.000	18	1330.584	12396302.000	19	1333.689	6027312.000	20	1334.723	2846790.000
21	1338.778	3421139.000	22	1346.580	33410280.000	23	1421.692	16644326.000	24	1437.685	2899053.000
25	1453.681	3269002.000	26	1475.753	2737019.000	27	1498.087	3651518.000	28	1514.063	2522535.000
29	1586.776	1920390.000	30	1628.761	6532752.000	31	1656.829	3284459.000	32	1705.947	12497191.000
33	1717.755	13796333.000	34	1754.807	3344757.000	35	1771.835	55665604.000	36	1793.818	1887480.000
37	1797.852	1901875.000	38	1813.844	3740478.000	39	1897.988	6251233.000	40	1922.978	7187925.000
41	1965.148	35370200.000	42	1993.982	3098879.000	43	2003.103	3494569.000	44	2039.020	3288979.000
45	2046.052	4111871.000	46	2150.087	1903918.000	47	2202.156	76594776.000	48	2234.147	38799428.000
49	2240.116	3195768.000	50	2272.104	3657977.000	51	2276.158	2058295.000	52	2316.083	56839276.000
53	2337.220	26418876.000	54	2358.091	2157787.000	55	2417.073	5551382.000	56	2420.213	16195770.000
57	2434.093	4767011.000	58	2500.281	10207933.000	59	2531.335	12331016.000	60	2568.307	9386221.000
61	2591.269	2700710.000	62	2606.218	16658981.000	63	2680.255	13401998.000	64	2789.349	17001606.000

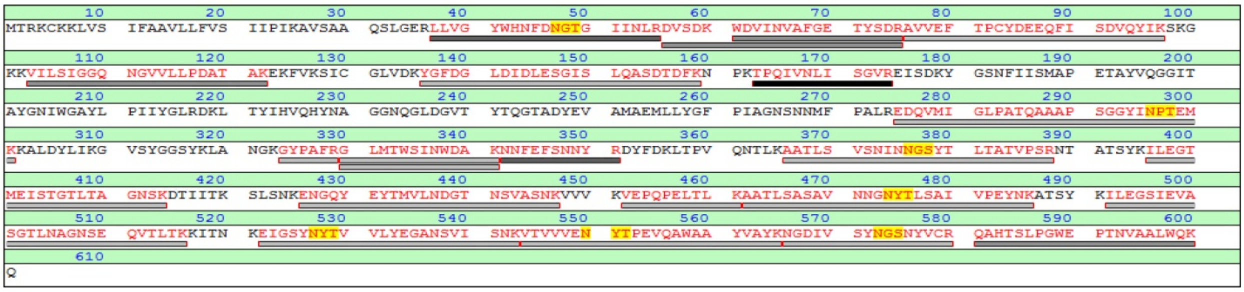

a)

Supplementary Figure S2. Peptide mass fingerprinting data for crChit62J4, original mass spectra (top), peak listing (middle), and sequence coverage of the translated open reading frame sequence peg.1890 – Chit62J4 from *Clostridium paraputrificum* J4 (bottom) are shown for four independent experiments (a-d). a) Intensity coverage 76.8%, sequence coverage 62.4%, b) Intensity coverage 77.6%, sequence coverage 59.9%, c) Intensity coverage 72.2%, sequence coverage 61.4%, d) Intensity coverage 76.3%, sequence coverage 63.9%.

(Supplementary Figure S2 cont.)

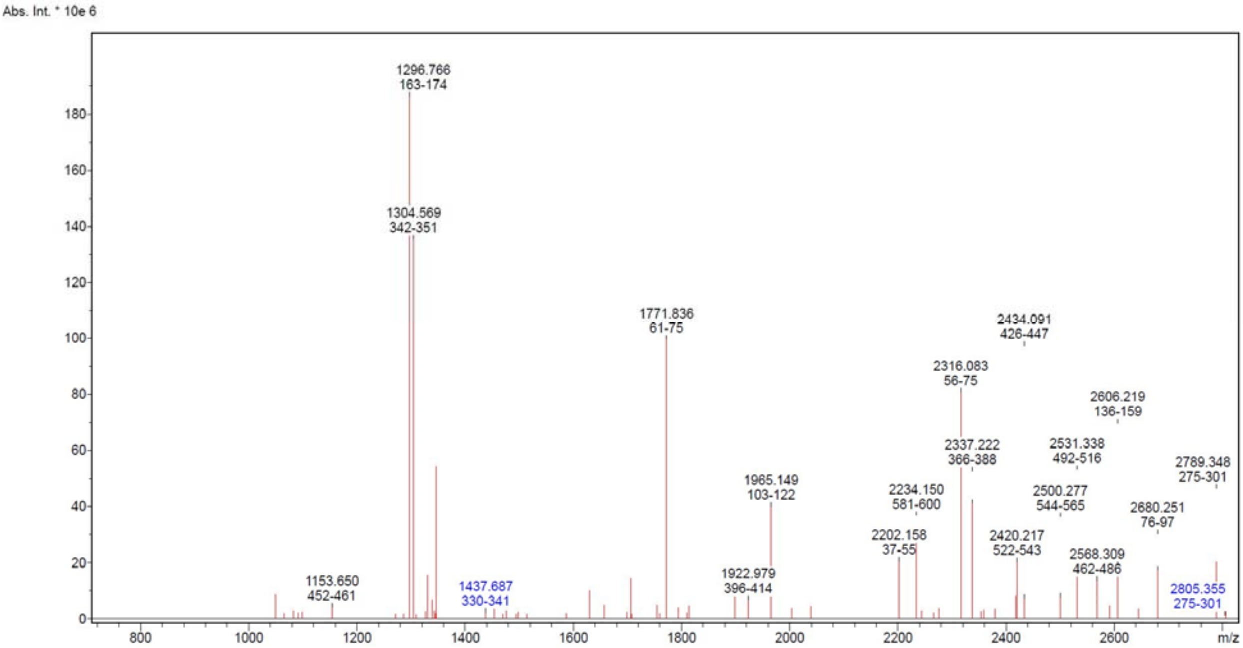

Peaklist:

Peak	Mass	Intensity	Peak	Mass	Intensity	Peak	Mass	Intensity	Peak	Mass	Intensity
1	710.364	6848090.000	2	1049.523	8610887.000	3	1065.517	2008359.000	4	1082.055	2959851.000
5	1091.533	2335327.000	6	1098.029	2400376.000	7	1153.650	5465113.000	8	1271.096	1694601.000
9	1286.511	1791920.000	10	1296.766	195551040.000	11	1304.569	136856128.000	12	1308.767	1509504.000
13	1326.553	2682095.000	14	1330.585	15598307.000	15	1338.780	6812801.000	16	1342.526	2882389.000
17	1344.563	1873440.000	18	1346.581	54215324.000	19	1437.687	3702261.000	20	1453.681	3672188.000
21	1469.675	1765062.000	22	1475.753	3068807.000	23	1493.740	1635457.000	24	1498.088	2494785.000
25	1514.062	1635610.000	26	1586.775	2000163.000	27	1629.758	10074081.000	28	1656.832	4796868.000
29	1698.756	2440267.000	30	1705.884	14294766.000	31	1707.778	1703686.000	32	1754.807	4759571.000
33	1759.775	1986927.000	34	1771.836	101009720.000	35	1793.820	3909396.000	36	1809.794	2164319.000
37	1813.845	4728578.000	38	1897.989	14638581.000	39	1922.979	8106374.000	40	1965.149	41403824.000
41	2003.107	3593643.000	42	2039.024	4441381.000	43	2202.158	22008016.000	44	2234.150	31649932.000
45	2244.168	2816936.000	46	2266.140	2253523.000	47	2276.160	3750625.000	48	2316.083	82096208.000
49	2337.222	41045552.000	50	2354.039	2611049.000	51	2358.094	3079903.000	52	2379.235	3378071.000
53	2417.077	8290636.000	54	2420.217	21765570.000	55	2434.091	7203117.000	56	2500.277	7645811.000
57	2531.338	19287078.000	58	2568.309	15048933.000	59	2591.277	4660953.000	60	2606.219	18842090.000
61	2644.191	3310311.000	62	2680.251	17339524.000	63	2789.348	20365824.000	64	2805.355	2681585.000
65	2830.389	1732475.000									

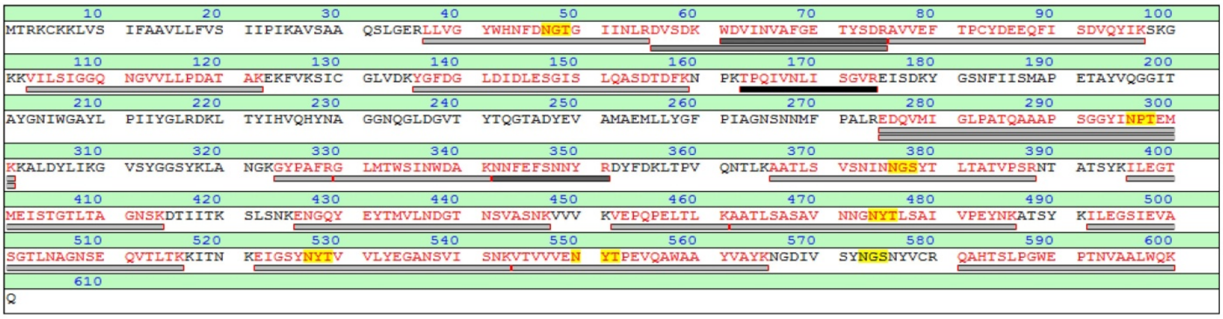

b)

(Supplementary Figure S2 cont.)

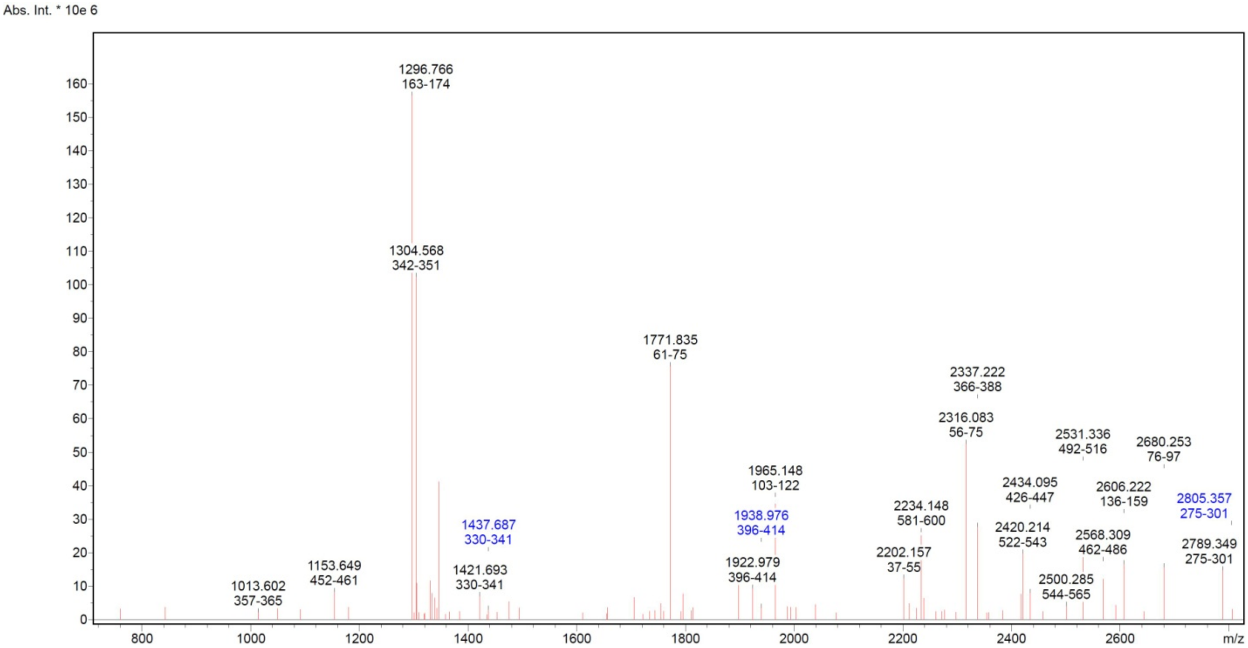

Peaklist:

Peak	Mass	Intensity	Peak	Mass	Intensity	Peak	Mass	Intensity	Peak	Mass	Intensity
1	710.363	11882500.000	2	760.396	3162115.000	3	842.511	3694052.000	4	1013.602	3369315.000
5	1049.523	7247788.000	6	1091.533	3058535.000	7	1153.649	9449314.000	8	1179.603	3711735.000
9	1296.766	164232016.000	10	1300.533	1992765.000	11	1304.568	103538296.000	12	1305.554	10957888.000
13	1309.055	2065579.000	14	1318.750	1703573.000	15	1320.587	1907478.000	16	1330.585	11571003.000
17	1333.689	7961566.000	18	1338.778	6553808.000	19	1342.525	3378451.000	20	1346.580	41213820.000
21	1358.578	1605469.000	22	1365.643	2347269.000	23	1383.694	2461790.000	24	1421.693	8193095.000
25	1434.775	1550488.000	26	1437.687	2933492.000	27	1453.679	2205167.000	28	1475.753	5358855.000
29	1493.740	3508810.000	30	1610.759	2029890.000	31	1654.789	1764708.000	32	1656.829	3586622.000
33	1705.852	6582473.000	34	1721.848	1582825.000	35	1733.878	2516234.000	36	1743.781	2772123.000
37	1754.807	4788264.000	38	1759.778	2527467.000	39	1771.835	76837464.000	40	1791.733	2456409.000
41	1794.822	7743606.000	42	1809.790	2688126.000	43	1813.845	3662924.000	44	1897.988	13431463.000
45	1922.979	10419443.000	46	1938.976	3577788.000	47	1965.148	34540452.000	48	1987.105	3927463.000
49	1993.982	3747975.000	50	2003.106	3664819.000	51	2039.023	4533479.000	52	2076.978	2046170.000
53	2202.157	13408848.000	54	2211.116	4832978.000	55	2225.125	3408449.000	56	2234.148	25100614.000
57	2239.147	6386153.000	58	2260.161	2395530.000	59	2272.099	2378942.000	60	2276.155	2938614.000
61	2297.210	2260328.000	62	2316.083	53662936.000	63	2337.222	27748904.000	64	2354.035	2017080.000
65	2358.096	2157302.000	66	2383.967	2737892.000	67	2417.072	7646348.000	68	2420.214	20912420.000
69	2434.095	7900921.000	70	2458.167	2386606.000	71	2500.285	5302698.000	72	2531.336	19007628.000
73	2568.309	12085400.000	74	2591.273	4284304.000	75	2606.222	16529814.000	76	2644.191	2446789.000
77	2680.253	15664976.000	78	2789.349	15905786.000	79	2805.357	3093646.000	80	2827.330	2444332.000

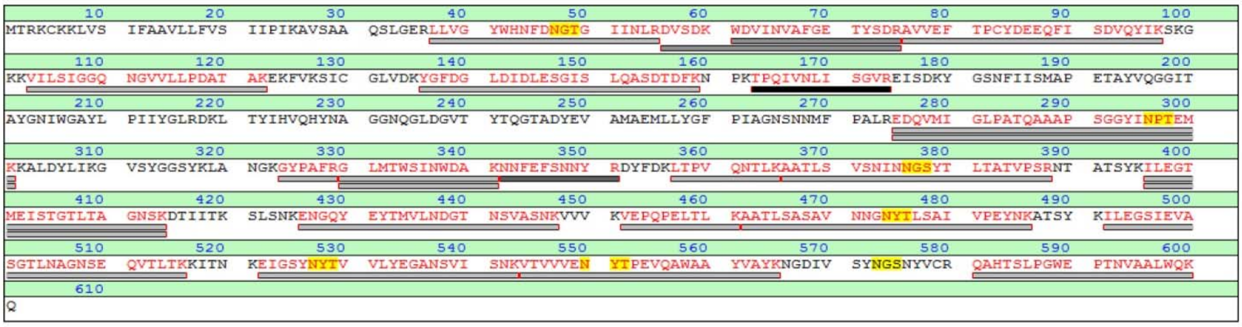

c)

(Supplementary Figure S2 cont.)

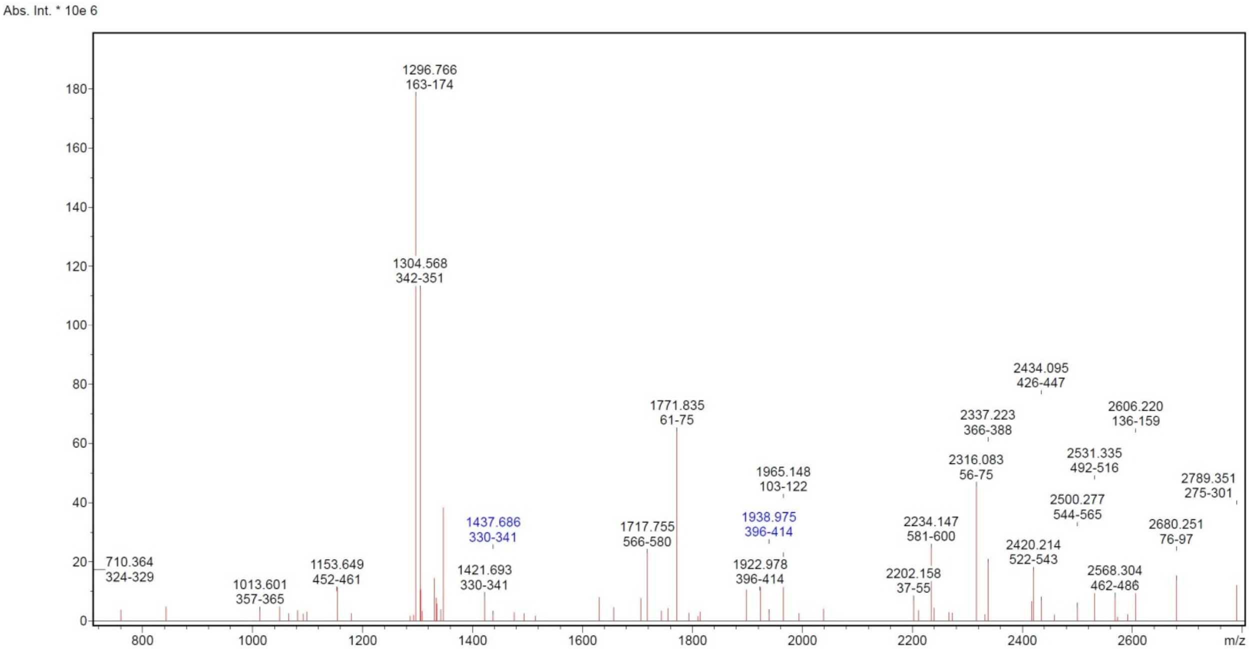

Peaklist:

Peak	Mass	Intensity	Peak	Mass	Intensity	Peak	Mass	Intensity	Peak	Mass	Intensity
1	710.364	12340896.000	2	760.396	3830154.000	3	842.511	4740910.000	4	1013.601	4819602.000
5	1049.522	8552011.000	6	1065.517	2638096.000	7	1082.055	3593344.000	8	1091.533	2411446.000
9	1098.028	2979181.000	10	1153.649	11514362.000	11	1179.603	2574783.000	12	1286.510	1714922.000
13	1293.079	2059209.000	14	1296.766	186389312.000	15	1304.568	113413616.000	16	1305.554	10600455.000
17	1309.053	3381451.000	18	1330.585	14396547.000	19	1333.689	7900010.000	20	1334.722	5845993.000
21	1342.526	3924459.000	22	1346.580	38261784.000	23	1421.693	9671189.000	24	1437.686	2145459.000
25	1475.753	2955225.000	26	1493.739	2479590.000	27	1514.062	1648013.000	28	1629.757	8003267.000
29	1656.830	4620969.000	30	1705.853	7591749.000	31	1717.755	24275192.000	32	1743.781	3388186.000
33	1754.808	4261807.000	34	1771.835	65352696.000	35	1793.818	2749753.000	36	1809.793	1475227.000
37	1813.842	3112028.000	38	1897.988	10553100.000	39	1922.978	11629520.000	40	1938.975	2620739.000
41	1965.148	21711482.000	42	1993.983	2572497.000	43	2039.023	4111928.000	44	2202.158	8493909.000
45	2211.113	3517304.000	46	2234.147	26083756.000	47	2239.147	4448765.000	48	2266.139	2896924.000
49	2272.105	2654972.000	50	2316.083	46922636.000	51	2332.085	2137308.000	52	2337.223	19681924.000
53	2417.071	6661793.000	54	2420.214	18274086.000	55	2434.095	6867112.000	56	2458.162	1975222.000
57	2500.277	4739723.000	58	2531.335	17976754.000	59	2568.304	9505688.000	60	2573.117	1294469.000
61	2591.263	2226033.000	62	2606.220	12630081.000	63	2680.251	13907604.000	64	2789.351	12038024.000
65	2805.353	2370028.000									

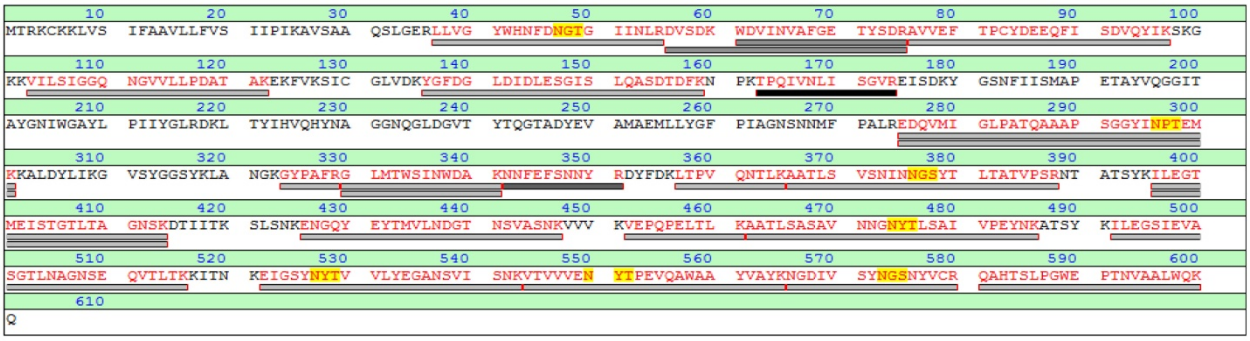

d)

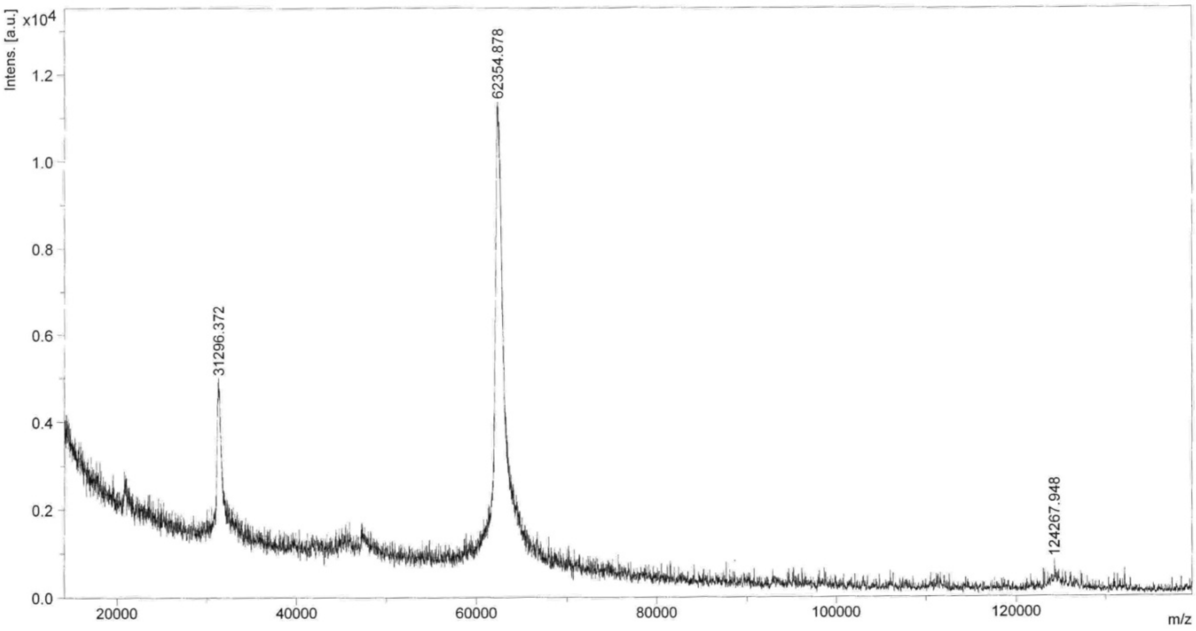

Supplementary Figure S3. MALDI-TOF spectrum of crChit62J4.

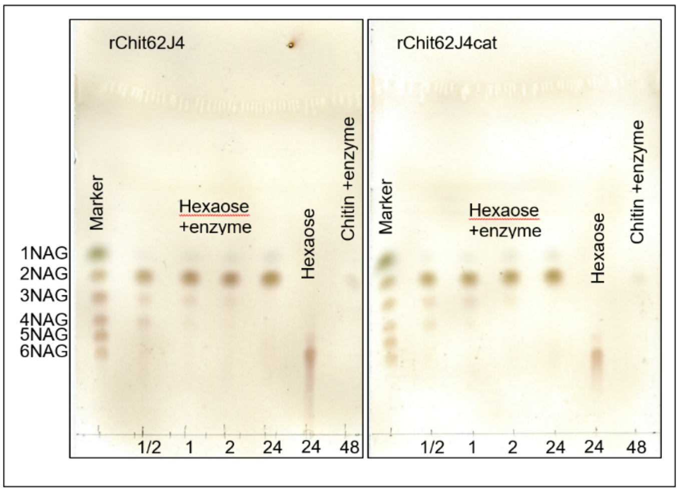

Supplementary Figure S4. Thin-layer chromatography analysis of reaction products of rChit62J4 (left panel) and rChit62J4cat (right panel) with chitinhexaose for ½ to 24 h and CM-chitin for 48 h (marked Chitin+enzyme). Marker is a mixture of N-acetyl glucosamine, chitinbiose, chitintriaose, chitintetraose, chitinpentaose and chitinhexaose in water marked as 1NAG, 2NAG, etc., respectively. Chitinhexaose was solubilized in the reaction mixture without the enzyme and applied as a control.

```
>ENA|KX353699|KX353699.1 Clostridium paraputrificum strain J4 chitinase Chit62 precursor,
gene, complete cds.
ATGACTAGAAAGTGTAAAAAACTAGTATCAATTTTTGCTGCTGTATTACTATTTGTATCA
ATTATTCCTATTAAGGCAGTTAGTGCAGCACAAAGCTTAGGAGAGAGGTTACTTGTGGGA
TATTGGCATAACTTTGATAATGGCACAGGAATTATTAACTTGAGGGATGTGTCAGATAAA
TGGGATGTTATAAACGTAGCTTTTGGAGAAACATATAGTGATAGAGCTGTAGTTGAATTT
ACACCTTGTTATGATGAAGAACAGTTTATTTCAGATGTTCAGTACATTAAGAGCAAAGGT
AAAAAAGTTATTTTATCAATAGGTGGACAAAATGGTGTAGTATTACTACCAGATGCAACA
GCTAAAGAAAAATTTGTTAAGTCAATATGTGGTTTAGTAGATAAATATGGCTTTGATGGT
TTGGATATAGACTTAGAATCCGGTATATCATTACAAGCATCAGATACAGATTTCAAGAAT
CCTAAAACACCACAAATAGTTAATCTTATTTCAGGTGTAAGGGAAATATCTGATAAATAT
GGATCAAACTTTATTATAAGTATGGCTCCAGAAACAGCTTATGTTCAAGGTGGAATTACT
GCTTATGGAAATATTTGGGGAGCTTATCTTCCAATAATATATGGATTAAGAGACAAATTA
ACTTATATACATGTACAACATTATAATGCTGGTGGTAATCAAGGATTAGATGGTGTCACA
TATACACAAGGAACAGCAGATTATGAAGTAGCAATGGCAGAAATGCTTTTATATGGATTT
CCTATAGCAGGTAATAGTAATAATATGTTCCCAGCTTTAAGAGAAGACCAAGTTATGATT
GGATTGCCAGCAACTCAGGCTGCTGCACCAAGTGGTGGATATATAAATCCAACAGAAATG
AAGAAAGCATTAGATTATTTAATTAAAGGTGTATCATATGGTGGAAGTTATAAGTTAGCT
AATGGTAAAGGATATCCAGCATTCAGAGGTTTAATGACATGGTCTATTAATTGGGATGCT
AAAAATAATTTTGAGTTTTCTAATAATTATAGAGATTACTTTGATAAACTAACTCCAGTT
CAAAATACATTAAAGGCTGCAACTCTTTCTGTTAGCAATATTAATAATGGAAGTTATACA
TTAACAGCTACAGTACCATCAAGAAATACTGCAACAAGTTATAAGATTCTAGAGGGGACA
ATGGAAATTTCTACAGGAACATTAACAGCAGGAAATTCTAAGGATACTATAATTACAAAG
AGTTTAAGCAATAAAGAAAATGGACAATATGAATATACTATGGTTTTAAATGATGGCACT
AATTCTGTAGCATCAAATAAGGTAGTAGTAAAGGTAGAACCTCAACCAGAACTTACATTA
AAAGCTGCAACATTATCAGCTAGCGCTGTTAATAATGGAAATTATACTCTATCAGCTATA
GTTCCTGAATATAATAAGGCAACAAGTTATAAAATTCTTGAAGGAAGTATAGAAGTAGCT
TCAGGTACATTAAATGCTGGTAATAGTGAACAAGTAACTCTTACAAAAAAAATCACAAAT
AAAGAGATTGGTAGCTATAATTACACAGTTGTTCTTTATGAAGGAGCAAATTCTGTTATA
TCAAATAAAGTAACTGTTGTAGTAGAAAACTACACACCAGAGGTCCAAGCTTGGGCAGCT
TATGTAGCATATAAAAATGGTGATATAGTAAGTTATAATGGAAGCAATTATGTTTGTAGA
CAGGCTCACACATCTTTACCAGGATGGGAGCCAACTAATGTAGCAGCATTATGGCAAAAA
CAATAG
```

a)

```
>tr|A0A1C9J7J5|A0A1C9J7J5_9CLOT Chitinase Chit62 OS=Clostridium
paraputrificum OX=29363 PE=3 SV=1
MTRKCKKLVSIFAAVLLFVSIIPIKAVSAAQSLGERLLVGYWHNFDNGTGIINLRDVSDK
WDVINVAFGETYSDRAVVEFTPCYDEEQFISDVQYIKSKGKKVILSIGGQNGVVLLPDAT
AKEKFVKSICGLVDKYGFDGLDIDLESGISLQASDTDFKNPKTPQIVNLISGVREISDKY
GSNFIISMAPETAYVQGGITAYGNIWGAYLPIIYGLRDKLTYIHVQHYNAGGNQGLDGVT
YTQGTADYEVAMAEMLLYGFPIAGNSNNMFPALREDQVMIGLPATQAAAPSGGYINPTEM
KKALDYLIKGVSYGGSYKLANGKGYPAFRGLMTWSINWDAKNNFEFSNNYRDYFDKLTPV
QNTLKAATLSVSNINNGSYTLTATVPSRNTATSYKILEGTMEISTGTLTAGNSKDTIITK
SLSNKENGQYEYTMVLNDGTNSVASNKVVVKVEPQPELTLKAATLSASAVNNGNYTLSAI
VPEYNKATSYKILEGSIEVASGTLNAGNSEQVTLTKKITNKEIGSYNYTVVLYEGANSVI
SNKVTVVVENYTPEVQAWAAYVAYKNGDIVSYNGSNYVCRQAHTSLPGWEPTNVAALWQK
Q
```

b)

Supplementary Figure S5. Sequence of Chit62J4. a) DNA sequence of the GeneBank record KX353699 and b) The translated amino acid sequence of the UniProt record A0A1C9J7J5.

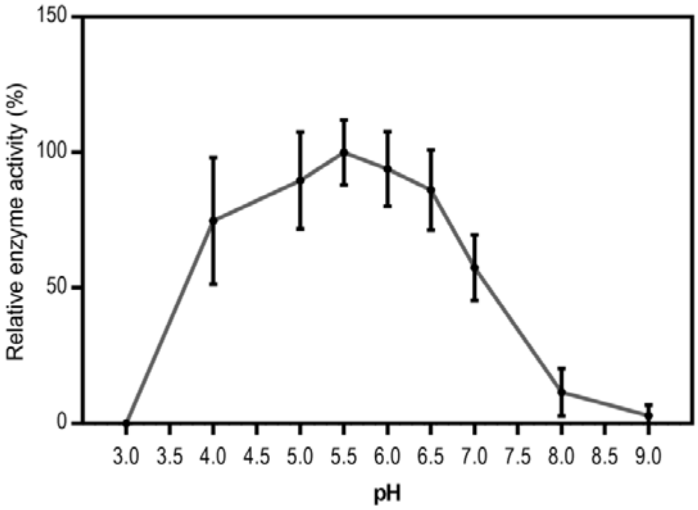

a)

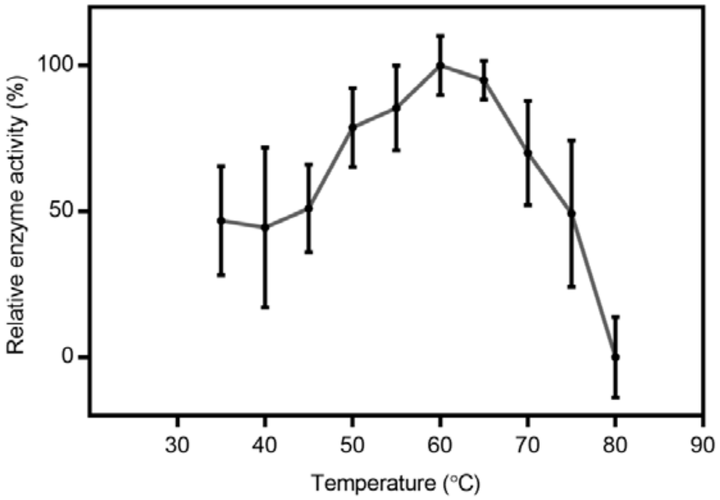

b)

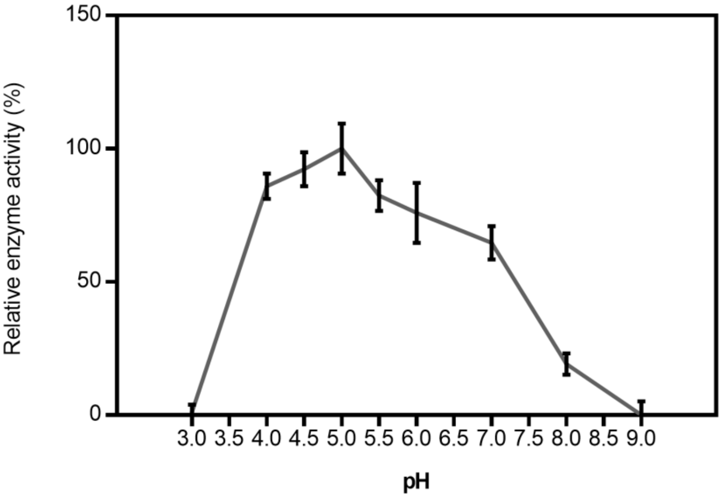

c)

Supplementary Figure S6. Dependence of the crChit62J4 activity toward pNGG on a) pH and b) temperature and c) of rChit62J4cat on pH. Mean values from three measurements and standard deviations are marked. Activities were normalized to the maximum value in the profile. Graphics created with GraphPad Prism version 7.02 for Windows (GraphPad Software, La Jolla California USA, www.graphpad.com).

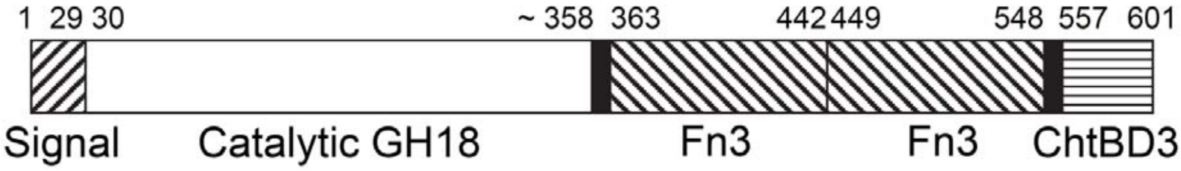

Supplementary Figure S7. Proposed domain structure of Chit62J4.

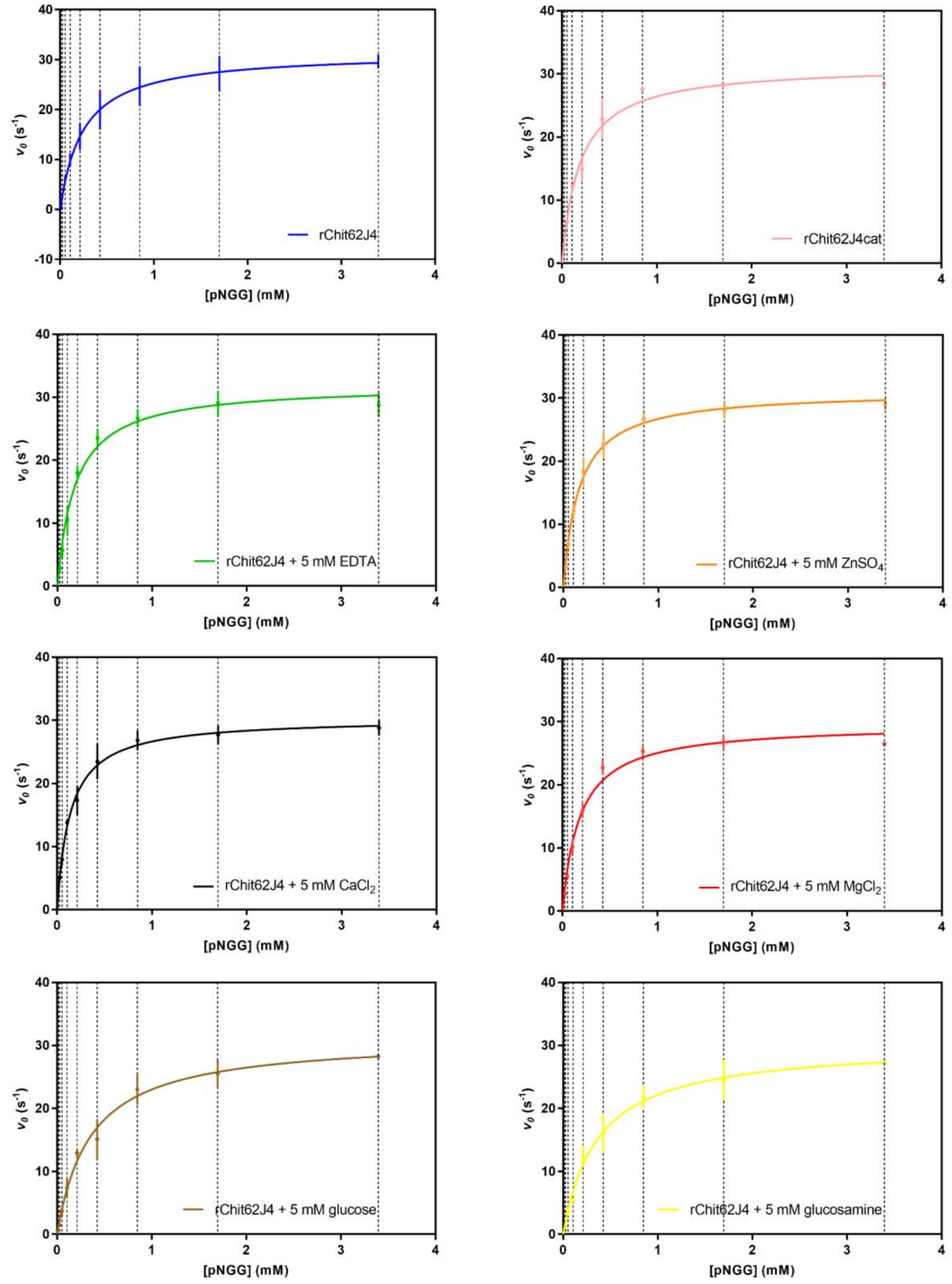

Supplementary Figure S8. Michaelis-Menten kinetics for rChit62J4 and rChit62J4cat and of rChit62J4 in the presence of selected compounds (marked in the legend). The activity was measured in triplicates for 9 concentration points (marked by vertical lines) and the mean value was used. Mean values and standard deviations are shown. The Michaelis-Menten curves were fitted using GraphPad Prism version 7.02 for Windows (GraphPad Software, La Jolla California USA, www.graphpad.com).

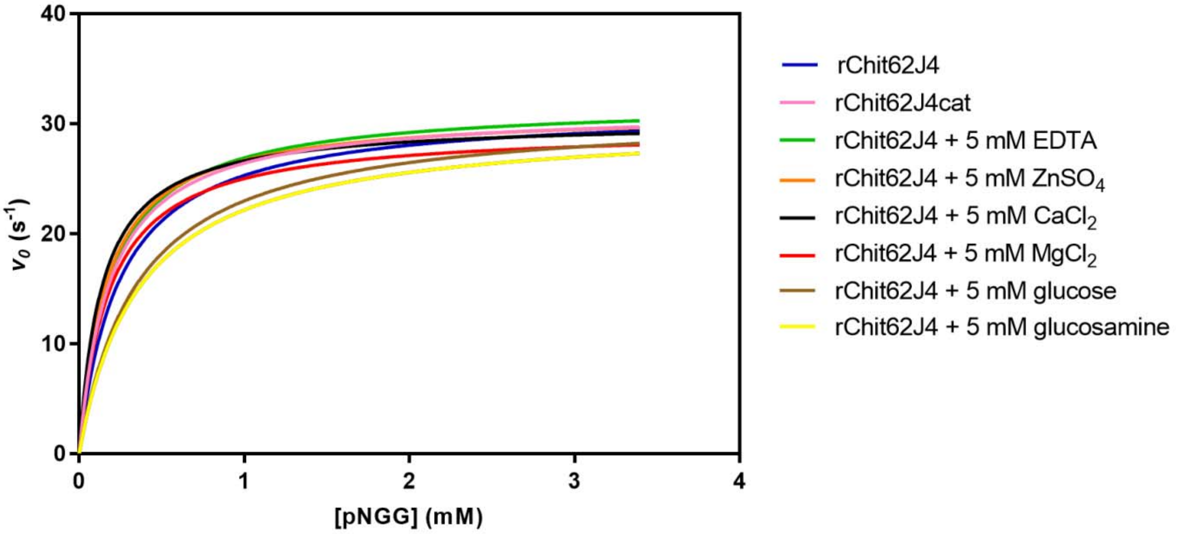

Supplementary Figure S9. Michaelis-Menten kinetics for rChit62J4 and rChit62J4cat and of rChit62J4 in the presence of selected compounds – an overlay of the resulting fitted curves from Supplementary Figure S6 (identical color coding is marked in the legend). The Michaelis-Menten curves were fitted using GraphPad Prism version 7.02 for Windows (GraphPad Software, La Jolla California USA, www.graphpad.com).

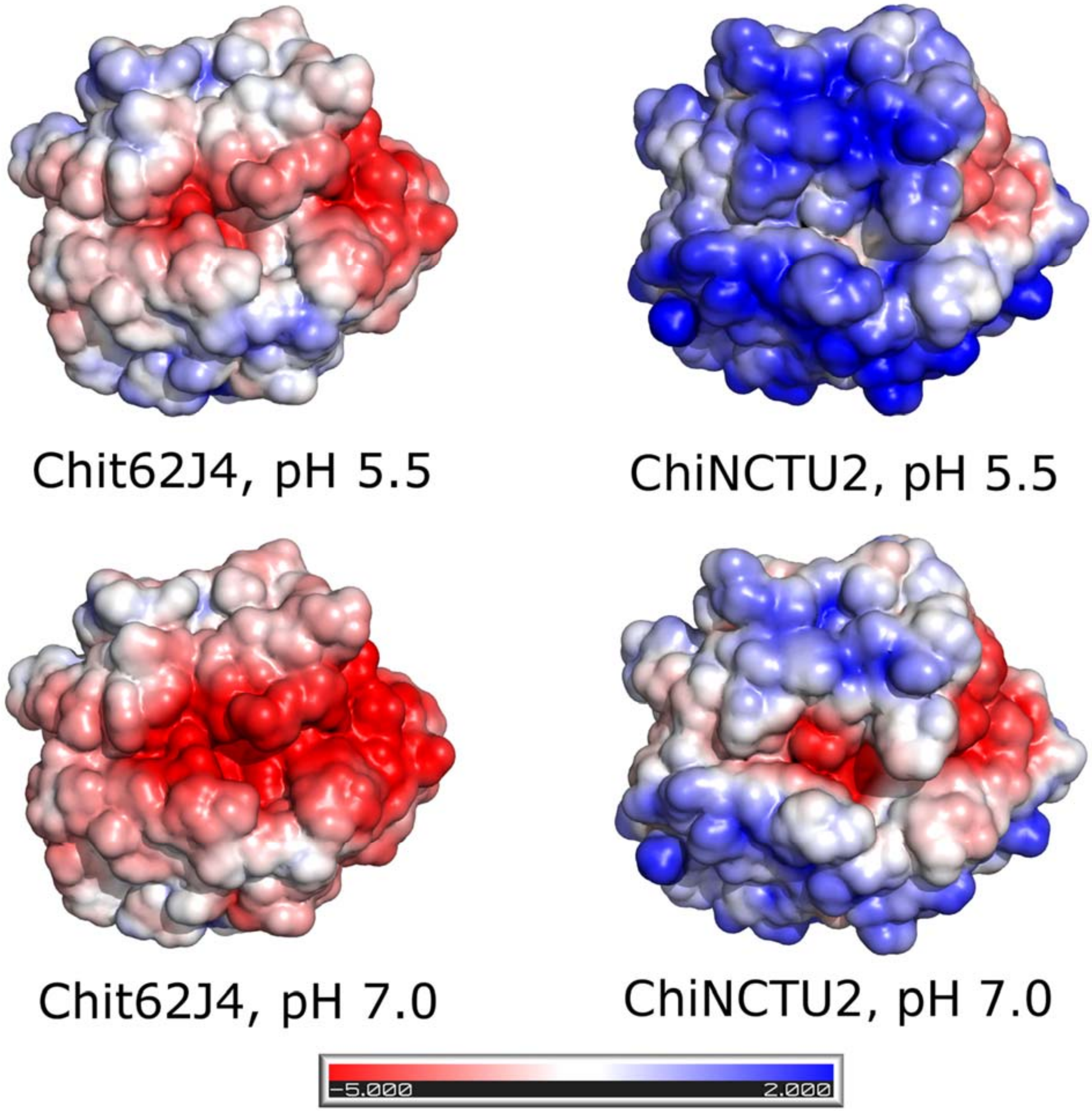

Supplementary Figure S10. Electrostatic surface potential of the catalytic domain of Chit62J4 (model) and chitinase ChiNCTU2 at important pH levels. Notice the similar behavior of the homologous enzymes at distinct pH levels following their pI points and showing enzyme optimization for function in different environments, Chit62J4 at pH 5.5 (pI 4.9) and ChiNCTU2 at pH 7 (pI 6.0). The color scale is set between electrostatic potential values –5 kT/e (red) and +2 kT/e (blue).

Table S1. Oligonucleotides applied for PCR amplification of rChit62J4 and rChit62J4cat.

Chit62J4pET15forw (XhoI)	TATATACTCGAGGCACAAAGCTTAGGAGAG
Chit62J4pET15rev (BamHI)	TATATAGGATCCCTATTGTTTTTGCCATAA
Chit62J4CATpET15rev (BamHI)	TATATAGGATCCTTAAACTGGAGTTAGTTT
